# Electrophysiological characterisation of motor and sensory tracts in patients with hereditary spastic paraplegia (HSP)

**DOI:** 10.1186/1750-1172-8-158

**Published:** 2013-10-09

**Authors:** Kathrin N Karle, Rebecca Schüle, Stephan Klebe, Susanne Otto, Christian Frischholz, Inga Liepelt-Scarfone, Ludger Schöls

**Affiliations:** 1 Department of Neurology, Eberhard Karls-University Tübingen, Tübingen 72076, Germany; 2 Hertie Institute for Clinical Brain Research, Eberhard Karls-University Tübingen, Tübingen, Germany; 3 German Research Center for Neurodegenerative Diseases (DZNE), Tübingen, Germany; 4 Department of Neurology, Christian-Albrechts-University Kiel, Kiel 24105, Germany; 5 Department of Neurology, Julius-Maximilians-University, Würzburg 97080, Germany; 6 Department of Neurology, St. Josef Hospital, Ruhr University, Bochum 44791, Germany

**Keywords:** Hereditary spastic paraplegia (HSP), Electrophysiology, Motor evoked potential (MEP), Somato-sensory evoked potential (SSEP), Neurography

## Abstract

**Background:**

Hereditary spastic paraplegias (HSPs) are characterised by lower limb spasticity due to degeneration of the corticospinal tract. We set out for an electrophysiological characterisation of motor and sensory tracts in patients with HSP.

**Methods:**

We clinically and electrophysiologically examined a cohort of 128 patients with genetically confirmed or clinically probable HSP. Motor evoked potentials (MEPs) to arms and legs, somato-sensory evoked potentials of median and tibial nerves, and nerve conduction studies of tibial, ulnar, sural, and radial nerves were assessed.

**Results:**

Whereas all patients showed clinical signs of spastic paraparesis, MEPs were normal in 27% of patients and revealed a broad spectrum with axonal or demyelinating features in the others. This heterogeneity can at least in part be explained by different underlying genotypes, hinting for distinct pathomechanisms in HSP subtypes. In the largest subgroup, *SPG4*, an axonal type of damage was evident. Comprehensive electrophysiological testing disclosed a more widespread affection of long fibre tracts involving peripheral nerves and the sensory system in 40%, respectively. Electrophysiological abnormalities correlated with the severity of clinical symptoms.

**Conclusions:**

Whereas HSP is primarily considered as an upper motoneuron disorder, our data suggest a more widespread affection of motor and sensory tracts in the central and peripheral nervous system as a common finding in HSP. The distribution patterns of electrophysiological abnormalities were associated with distinct HSP genotypes and could reflect different underlying pathomechanisms. Electrophysiological measures are independent of symptomatic treatment and may therefore serve as a reliable biomarker in upcoming HSP trials.

## Background

Hereditary spastic paraplegias (HSPs) encompass a group of neurodegenerative disorders with lower limb spasticity due to degeneration of the corticospinal tract as most prominent sign. In addition to this “pure” form, additional neurological and non-neurological symptoms, such as mental retardation, dementia, epilepsy, cerebellar signs, extrapyramidal symptoms, sensory deficits, peripheral neuropathy, skin and skeletal abnormalities can be present (“complicated” forms) [1-4]. HSP is a rare disease with a prevalence of ~ 2–10: 100,000 inhabitants [5,6]. To date, more than 50 loci (SPG1 – SPG57) and more than 30 genes have been described [7].

Electrophysiological features of HSP have been studied in rather small cohorts (maximum of 26 patients per study) with poor genetic characterisation. Motor evoked potentials were found to be abnormal to the legs in the majority of patients and to the arms in up to one third of patients [8-11]. Central and peripheral sensory tracts were affected to very variable degrees [9,12-14]. First results hint to genotype-related changes in transcranial magnetic stimulation in the largest HSP subgroup, *SPG4*[15,16].

To analyse the spread of long fibre tract affection in HSP and to explore potential effects of different pathomechanisms in distinct genotypes we studied motor and sensory involvement of the central and peripheral nervous system by clinical and electrophysiological means in a representative cohort of HSP patients.

## Methods

### Patients

128 patients (58 women, 70 men) from 109 families were recruited by specialised HSP outpatient clinics in Bochum, Kiel, and Tübingen, Germany, in the context of the German Network of Hereditary Movement Disorders (GeNeMove). Diagnostic criteria for HSP included (i) spastic paraparesis or spastic tetraparesis with legs earlier and more severely affected than arms or (ii) spastic paraparesis as early and prominent sign of a neurodegenerative multisystem disease after exclusion of other causes. To exclude secondary forms of spastic paraparesis standard diagnostic procedures covered MRI of head and spine, vitamin B12 and folic acid levels, very long chain fatty acids (VLCFA), neurometabolic screening (Krabbe disease, metachromatic leukodystrophy, GM1-gangliosidosis, GM2-gangliosidoses Tay Sachs and Sandhoff, Gaucher disease) and cerebrospinal fluid analysis. All participants gave their written informed consent. The study was approved by the ethics committee of the recruiting centres.

Mean age of patients at examination was 47.5 ± 14.9 years (13–80 years). Disease onset varied between 0 and 68 years (mean age at onset 29.6 ± 17.3 years), mean disease duration was 18.0 ± 13.1 years (0–64 years). At examination 35 patients used a walking aid, 15 patients were wheelchair-bound.

Family history was positive in 55 of 109 families, including 46 families with autosomal dominant (42%) and 9 families with autosomal recessive (8%) disease inheritance. The diagnosis was genetically confirmed in 54 of 128 cases (46%). In addition to 35 cases with *SPG4* the following genotypes were identified: *SPG3* (1), *SPG5* (3), *SPG7* (3), *SPG8* (1), *SPG10* (1), *SPG11* (6), and *SPG15* (4). *SPG4* mutations were excluded in 34 of the 74 cases with unknown genotype (“*non-SPG4* patients”).

Clinical severity was assessed by Spastic Paraplegia Rating Scale (SPRS) [17]. The sum of the point values for spasticity of hip adductor muscles, spasticity of knee flexion, weakness of hip abduction, and weakness of foot dorsiflexion were designated as “spastic subscore”. Pure HSP was diagnosed if spastic paresis was accompanied by impaired vibration sense and/or urinary urgency only (56% of patients in our cohort). Presence of other additional signs or symptoms resulted in classification as complicated HSP (44% of our patients) [2]. Peripheral motor neuropathy was clinically assumed by summing up the number of the following items: loss of muscle stretch reflexes to the upper limbs, loss of muscle stretch reflexes to the lower limbs, muscle wasting of the upper limbs, and muscle wasting of the lower limbs. Analogously, the sensory system was rated to be clinically affected (“clinical sensory deficit”) by summing up the number of the following affected parameters: touch sense, pinprick sensation, vibration sensation (< 6/8), joint position sense, and temperature discrimination.

### Electrophysiological techniques

Motor evoked potentials (MEPs) were measured to the right and left abductor digiti minimi (ADM) and abductor hallucis (AH) muscle after muscle activation. For total motor conduction time (TMCT) stimulus was given with a circular coil over the vertex and stimulus intensity was chosen 20% above motor threshold at rest. Peripheral motor conduction time (PMCT) was calculated with the shortest F wave latency out of 16 trials after supramaximal stimulation of the ulnar nerve at the wrist or the tibial nerve at the medial malleolus respectively: PMCT = [(distal motor latency + F wave latency)/2] +1. Central motor conduction time CMCT = TMCT – PMCT.

Nerve conduction studies (NCS) with surface electrodes were performed on the right side, except local problems would have falsified the results. Motor NCS were performed of the ulnar and tibial nerve. Amplitudes of compound motor action potentials (CMAPs) were measured peak to peak. Stimulus intensity was increased in 5 mA steps to maximum response. Stimulus duration was adjusted 0.2 ms, and increased, if supramaximal stimulation was not reached otherwise. Recording electrode was placed over the abductor digiti minimi and abductor hallucis muscle respectively, distal stimulation was performed with the electrode 5 and 7 cm proximal to the recording electrode and proximal stimulation with the electrode at the elbow distal the sulcus ulnaris and at the popliteal fossa respectively.

Sensory NCS were recorded from the radial and sural nerve with antidrome technique and supramaximal stimulus intensity. Amplitudes of sensory nerve action potentials (SNAPs) were measured baseline to peak. The recording electrode was placed over the first dorsal spatium interosseum and behind the external malleolus respectively, stimulation electrode 15 cm and 14 cm proximal to the recording electrode respectively. Electrophysiologically peripheral neuropathy was assumed, if NCS were abnormal in two or more nerves.

Somato-sensory evoked potentials (SSEPs) of median and tibial nerve were acquired using surface electrodes over C3’/C4’ and Cz’ (3 cm posterior to Cz) respectively, and referred to Fpz. Electrode impedances were less than 5 kOhm. The constant current stimuli were rectangular electrical pulses of 0.2 ms duration, delivered 3 times per second at the medial side of the ankle and the wrist, respectively. Stimulus intensity was adjusted to produce a small muscle twitch. Overall bandpass was chosen 1 to 1500 Hz, analysis time was 100 ms. Two series of 200 potentials were recorded, averaged, superimposed, and checked for reproducibility. The latency was measured at Erb and N20 for median nerve, and L1 and P40 for tibial nerve.

### Normal values and statistical analysis

Standard procedures and normal values were elaborated for NCS, MEP and SSEP in Tübingen, and transferred to the centres in Bochum and Kiel. Extreme values were checked for plausibility. Statistical analyses were performed using IBM SPSS Statistics 20.0 software (IBM Deutschland GmbH, Ehningen, Germany). Mean and standard deviation are given. Parameters were either normally distributed or slightly skewed (< 3.12), therefore parametric testing was applied. For continuous variables bivariate correlations were performed using the Pearson correlation coefficient, for categorised variables using Kendalls Tau-b. Significance was tested by one- or two-sided ANOVA, as appropriate. An alpha level of p < 0.05 was considered to be significant.

## Results

### Clinical characterisation

In our cohort, disease severity assessed by SPRS reached 1 to 44 points (mean 17.8 ± 8.5 points); the spastic items (“spastic subscore”, as described in Methods section) ranged from 0 to 14 points (mean 4.6 ± 2.5 points). A clinical sensory deficit was obvious in 56% of patients (including patients with only vibration sense deficits), a peripheral motor neuropathy was clinically suspected in 18% of patients, and 22% of patients had upper limb spasticity in addition to spastic paraparesis.

### Central motor pathways

**Figure 1:**
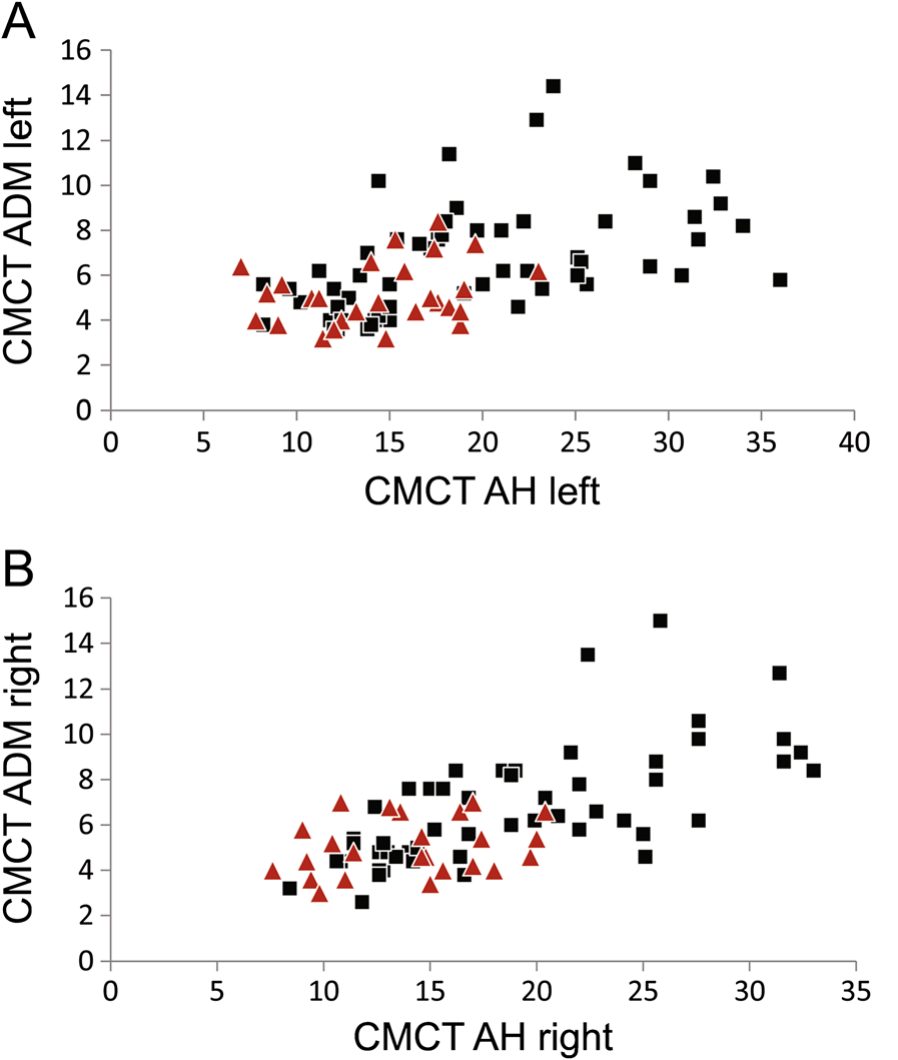
**Correlation of central motor conduction time (CMCT) to arm and leg.** CMCT to the abductor digiti minimi muscle (ADM) and abductor hallucis muscle (AH) is correlated (r = 0.595; p < 0.0001). In **(A)** results for the left-hand side, in **(B)** for the right-hand side are shown. Patients with *SPG4* mutations are indicated by red triangles, other patients by black squares.

CMCT to upper and lower limbs correlated with each other (r = 0.595; p < 0.0001; see also Figure 1). Age had no influence on CMCT.

**Figure 2:**
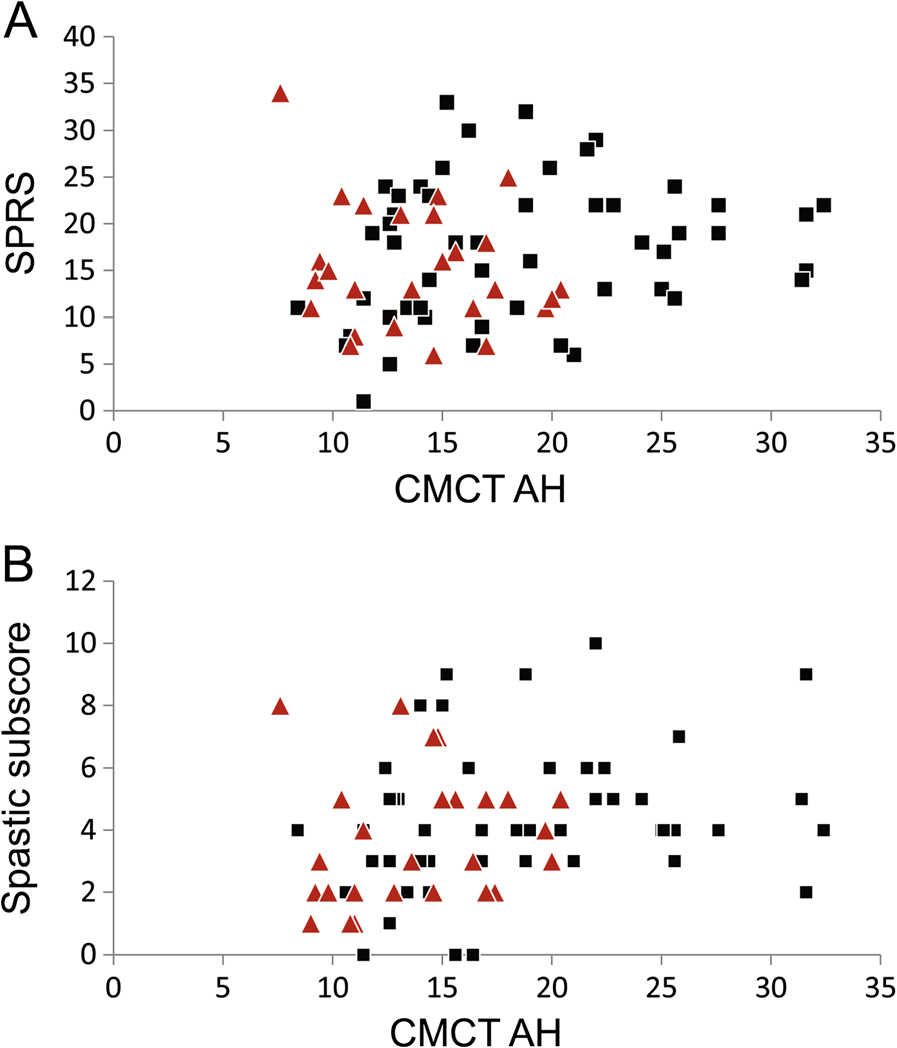
**Conduction in motor evoked potentials correlate with clinical disease severity.** In **(A)** Spastic Paraplegia Rating Scale (SPRS) [17], in **(B)** spastic subscore is correlated with central motor conduction time (CMCT) to the right abductor hallucis muscle (AH) (SPRS: r = 0.176; p < 0.028. Spastic subscore: r = 0.241, p < 0.005). Patients with *SPG4* mutations are indicated by red triangles, other patients by black squares. Results to the left leg correlated accordingly; data not shown.

Although pyramidal tract affection to the legs is an obligatory feature and the primary hallmark of HSP, motor evoked potentials (MEPs) to the legs were normal in 27% of patients. 37% presented with prolonged CMCT. In 36% of patients no MEP could be evoked. CMCT to the legs correlated with total SPRS score (r = 0.176; p < 0.028) and spastic subscore (r = 0.241, p < 0.005, see also Figure 2). Pathologic CMCT to lower limbs correlated with disease duration (r = 0.231; p < 0.009).

In contrast, MEPs to the arms were surprisingly often pathologic in HSP (32%), including 28% with prolonged and 4% with not evocable MEPs. CMCT to the arms correlated with total SPRS score (r = 0.234, p < 0.005) and spastic subscore (r = 0.300, p < 0.0001), but not with disease duration.

A subgroup of seven patients (6%) showed very pronounced CMCT prolongation to the arms (≥ 15.0 ms, normal < 8.6 ms), and 17 (14%) to the legs (≥ 25.0 ms, normal < 16.1 ms) suggestive of a demyelinating process. Most of the patients were of unknown genotype, in two patients a mutation was found in the *SPG5* and *SPG7* gene respectively. In none of these patients a *SPG4* mutation was found.

### Sensory pathways

SSEPs were available for median (n=47) and tibial (n=86) nerve stimulation. As peripheral stimulation at Erb and L1 was not available in several patients due to spastic reactions to repetitive stimulation, we included only cortical latencies in the analysis. Cortical latencies (N20) of median nerve SSEPs were prolonged in 9% and missing in 9%. Cortical latencies (P40) of tibial nerve SSEPs were delayed in 7% and missing in 29%. P40 latencies correlated with clinical sensory deficit (r = 0.314; p < 0.001), whereas for N20 latency significance was missed (r = 0.264; p < 0.063). Sensory nerve conduction velocities (SNCVs) correlated with cortical SSEP latencies in upper and lower limbs: Radial NCV correlated with N20 latency (r = 0.521; p < 0.003) and sural NCV with P40 latency (r = 0.326; p < 0.001). The validity of the correlation of SNCV and SSEP is limited as cortical latencies of SSEP also include peripheral nerve conduction time.

### Peripheral nerve involvement

NCS were abnormal in 75 patients (59%). In 17% of patients only sensory nerves, in 16% only motor nerves and in 25% both sensory and motor nerves were affected. In 15% of patients only legs were affected, in 14% only arms, and in 30% of patients both legs and arms were involved. Details of nerve conduction abnormalities are given in Table 1.

**Table 1:** Peripheral nerve involvement assessed by nerve conduction studies (NCS) in HSP patients

**Nerve**	**N**	**Percentage of patients with abnormal**				
		**NCS**	**DML**	**NCV**	**Amplitude**	**F wave latency**
Motor tibial nerve	128	33.9%	17.2%	22.7%	10.2%	8.7%
Motor ulnar nerve	128	23.8%	11.8%	4.7%	0.8%	15.9%
Sensory sural nerve	128	24.2%	-	21.9%	14.8%	-
Sensory radial nerve	100	36.0%	-	17.0%	31.0%	-

N: number of patients examined, DML: distal motor latency, NCV: nerve conduction velocity.

Abnormal distal motor latency (DML), NCV or F wave latency in combination with normal amplitudes were regarded as hint for demyelinating affection, whereas amplitude reduction and normal conduction parameters were interpreted as axonal involvement. According to this classification, 24% of patients presented with demyelinating, 5% with axonal and 6% with mixed neuropathy of tibial nerves, whereas in 66% neurography of tibial nerve was normal. In ulnar nerve 23% of patients showed a demyelinating and 1% a mixed pattern. In sural nerves, type of damage was demyelinating in 9% of patients, axonal in 9%, and mixed in 6%. In radial nerves 5% of patients presented with demyelinating and 18% with axonal type of damage, and 12% a mixed affection.

**Figure 3:**
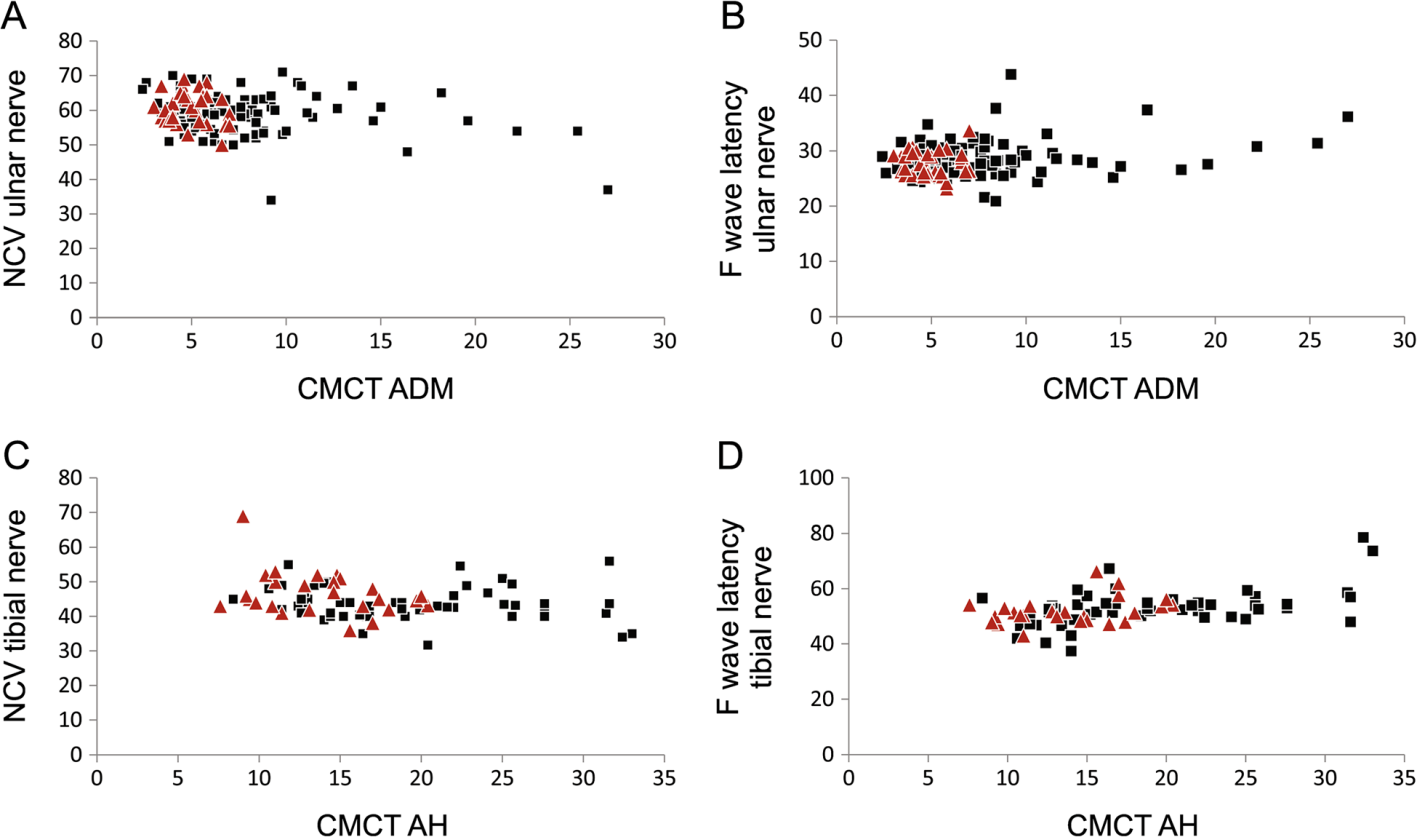
**Correlation of central and peripheral motor damage. (A and B)** Nerve conduction velocity (NCV) and F wave latency of the ulnar nerve correlated with central motor conduction time (CMCT) to the abductor digiti minimi muscle (ADM) (r = 0.249; p < 0.004 and r = −0.280; p < 0.002, respectively). **(C and D)** Accordingly, NCV and F wave latency of tibial the nerve correlated with CMCT to abductor hallucis muscle (AH) (r = 0.463; p < 0.0001 and r = −0.270; p < 0.09, respectively). Patients with *SPG4* mutations are indicated by red triangles, other patients by black squares.

Amplitudes of CMAPs and SNAPs were inversely correlated with age, so the correlations of NCS were analysed with age as confounding factor. Central and peripheral conduction velocities correlated both in upper and lower limbs (Figure 3): NCV and F wave latency of the ulnar nerve correlated with CMCT to the arm (r = 0.249; p < 0.004 and r = −0.280; p < 0.002, respectively). Accordingly, NCV and F wave latency of the tibial nerve correlated with CMCT to the leg (r = 0.463; p < 0.0001 and r = −0.270; p < 0.09, respectively).

Clinical signs of peripheral nerve involvement correlated with electrophysiological peripheral neuropathy (r = 0.362; p < 0.0001). Clinical sensory deficits correlated with pathologic NCS of the sural nerve (r = 0.291; p < 0.004).

### Subgroup analysis of genetically confirmed HSP patients

#### SPG4 patients

A clinically “pure” HSP form was found more often in *SPG4* patients in comparison to patients with *SPG4* excluded (*non-SPG4* patients) (60% *vs.* 36%, p = 0.052). Nystagmus, limb ataxia, gait ataxia, impaired pinprick sensation, impaired joint position sense, and clinical signs for spasticity of the arms were significantly rarer in *SPG4* patients (p < 0.05, respectively).

In most *SPG4* patients CMCT was normal both to arms (mean 5.1 ± 1.5 ms) and legs (14.1 ± 3.9 ms). In contrast, *non-SPG4* patients presented with significantly longer CMCT to arms (mean 6.9 ± 2.6 ms, p < 0.001) and legs (mean 18.2 ± 7.3 ms, p < 0.016; see also Figures 1 and 2). Within the group of *SPG4* patients, CMCT depended on the type of mutation. Patients with *SPG4* missense mutations had significantly shorter CMCTs to legs in comparison to patients with *SPG4* splice site mutations, premature stop codon or in-frame exon deletions (10.5 ± 1.3 ms *vs.* 13.9 ± 3.7 ms, p < 0.013). In *SPG4* patients nerve conduction abnormalities of tibial and ulnar motor nerves were present in 20.0% and 8.8% respectively. Sensory sural and radial nerves were pathologic in 20.0% and 24.1% respectively.

#### Other genetically defined HSP forms

Genetic testing revealed 19 patients with mutations in HSP genes other than *SPG4* (see Methods section). In the majority of these patients CMCT to the legs was prolonged. Details of motor evoked potentials in different genotypes are given in Table 2. Nerve conduction studies were normal in the single *SPG3* patient whereas *SPG5*, SP*G8* and *SPG10* patients presented with mild motor neuropathy and patients with *SPG11* and *SPG15* had sensory-motor neuropathy. In *SPG7* patients sensory tracts were affected. Detailed results of electrophysiological testing are presented in Table 3.

**Table 2:** Transcranial magnetic stimulation and sensory evoked potentials in genetically defined subtypes of HSP

**Genotype**	**CMCT ADM (in ms)**	**CMCT AH (in ms)**	**N20 latency (in ms)**	**P40 latency (in ms)**
*SPG3*	0/1	1/1	n.d.	n.d.
	6.4	29.0		
*SPG4*	0/35	17/35	0/12	6/24
	5.1 ± 1.4	14.1 ± 3.9	19.9 ± 1.1	42.6 ± 3.6
	3.0-8.4	7.0-23.0	18.0-21.8	37.8-52.6
*SPG5*	1/3	3/3	2/2	2/2
	7.8 ± 2.5	24.4 ± 5.5	-	-
	5.2-10.6	19.0-29.0	-	-
*SPG7*	0/3	2/3	0/1	1/2
	4.4 ± 1.5	18.6 ± 6.5	20.9	46.3 ± 3.0
	2.4-6.4	13.8-25.6	-	42.8-49.6
*SPG8*	1/1	1/1	0/1	1/1
	-	-	19.3	49.7
*SPG10*	0/1	1/1	0/1	1/1
	3.9	-	19.0	-
*SPG11*	1/6	4/6	0/2	2/4
	4.9 ± 1.0	13.3 ± 1.4	20.0 ± 0.6	43.2 ± 0.7
	3.6-6.0	12.0-15.2	19.0-20.5	42.0-44.6
*SPG15*	3/4	4/4	n.d.	1/1
	7.6 ± 2.4	-		50.8
	4.4-10.2	-		-
Threshold value	< 8.6	< 16.0	< 23.3	< 49.0

For each genotype is given: in the first row the number of pathologic results in the number of individuals examined, in the second row mean and standard deviation, and in the third row the range of values. CMCT: central motor conduction time, ADM: abductor digiti minimi, AH: abductor hallucis, N20: primary cortical negativity of somatosensory potentials with median nerve stimulation, P40: primary cortical positivity of somatosensory potentials with tibial nerve stimulation, n.d.: not done.

**Table 3:** Nerve conduction studies in genetically defined subtypes of HSP

**Genotype**	**Tibial nerve**				**Ulnar nerve**				**Sural nerve**		**Radial nerve**	
	**DML (in ms)**	**CMAP (in mV)**	**MNCV (in m/s)**	**FWL (in ms)**	**DML (in ms)**	**CMAP (in mV)**	**MNCV (in m/s)**	**FWL (in ms)**	**SNAP (in μV)**	**SNCV (in m/s)**	**SNAP (in μV)**	**SNCV (in m/s)**
*SPG3*	0/1	0/1	0/1	0/1	0/1	0/1	0/1	0/1	0/1	0/1	0/1	0/1
	3.6	14.6	52	48.8	2.2	10.1	61	28	24.0	57	20.3	57
*SPG4*	6/35	0/35	2/35	1/35	1/34	0/34	1/34	1/34	4/35	6/35	5/29	2/29
	4.3±0.8	18.7±6.8	47.2±5.7	51.7±4.9	2.4±0.4	15.9±3.2	60.8±4.5	27.6±2.3	11.1±7.8	47.7±7.0	22.2±7.0	62.0±5.0
	3.0-6.5	7.3-35.0	36-69	43.0-66.3	1.7-3.2	6.8-22.7	50-69	23.2-33.7	0–37.7	34-64	10.9-35.2	50-73
*SPG5*	0/3	0/3	0/3	0/3	1/3	0/3	0/3	0/3	0/3	0/3	0/3	0/3
	3.8±0.9	26.6±11.0	46.0±5.0	48.7±6.5	2.8±0.6	15.5±4.4	66.7±3.2	24.3±2.7	28.7±13.1	46.7±8.1	32.9±2.6	64.3±4.2
	2.9-4.7	17.3-38.7	41-51	41.2-53.0	2.1-3.3	12.8-20.5	63-69	21.6-27.0	14.6-40.5	41-56	30.6-35.7	61-69
*SPG7*	0/3	0/3	0/3	0/3	0/3	0/3	0/3	0/3	0/3	0/3	0/3	0/3
	4.0±0.4	22.1±9.1	46.7±3.5	47.5±5.7	2.2±0.3	15.1±4.0	65.7±1.5	28.8±0.3	17.5±4.9	48.7±2.3	24.0±2.4	63.7±2.5
	3.7-4.5	11.7-28.3	43-50	41.2-52.4	1.8-2.4	12.3-19.7	64-67	28.4-29.0	12.9-22.6	46-50	21.9-26.6	61-66
*SPG8*	0/1	0/1	1/1	0/1	0/1	0/1	0/1	0/1	0/1	0/1	0/1	0/1
	3.2	10.2	37	54.3	2.4	16.1	59	28.3	26.0	43	19.7	59
*SPG10*	0/1	0/1	1/1	0/1	0/1	0/1	0/1	0/1	0/1	0/1	0/1	0/1
	4.2	22.1	40	51.4	2.5	11.4	51	27.2	5.9	52	23.1	61
*SPG11*	1/6	1/6	3/6	1/5	1/6	2/6	0/6	1/5	1/6	1/6	2/5	3/5
	4.7±2.1	18.6±12.2	41.7±3.6	51.9±7.6	2.9±0.7	14.0±7.1	61.5±4.3	27.6±2.0	12.1±10.9	47.0±5.8	19.2±5.6	55.0±4.4
	3.6-8.8	0.9-36.1	37-46	45.1-64.8	2.1-4.3	1.1-21.1	58-70	24.6-29.0	1.6-31.6	38-56	12.5-24.5	48-60
*SPG15*	0/4	0/4	3/4	0/4	0/4	0/4	0/4	2/4	1/4	1/4	1/3	1/3
	4.3±1.0	8.8±3.8	38.3±5.7	56.9±5.4	2.7±0.2	14.2±2.3	55.3±4.6	30.3±2.8	7.1±3.7	47.5±13.9	18.6±6.0	56.3±2.9
	2.8-5.0	5.8-14.3	30-43	49.0-61.0	2.4-2.8	11.6-16.4	51-61	26.1-32.2	2.9-11.9	31-65	11.7-22.4	53-58
Threshold value	< 5.1	> 5.0	> 40	< 63.6	< 3.2	> 4.0	> 50	< 31.0	> 3.8	> 39	> 16.0	> 55

For each genotype is given: in the first row the number of pathologic results in the number of individuals examined, in the second row mean and standard deviation, and in the third row the range of values. DML: distal motor latency, CMAP: compound motor action potential, MNCV: mean nerve conduction velocity, FWL: F wave latency, SNAP: sensory nerve action potential, SNCV: sensory nerve conduction velocity.

## Discussion

Comprehensive electrophysiological analyses in this up to date largest cohort of HSP patients revealed not only prominent affection of corticospinal tracts to the legs but also in many cases to the arms as well as affection of sensory systems and peripheral nerves. This affirms that HSP is not a pure upper motoneuron disease but frequently affects other long fibre tracts.

Some authors regard spread of corticospinal tract affection to the arms as an indicator of primary lateral sclerosis rather than HSP [18]. Here we show that according to MEP criteria arms are involved in about one third of HSP patients including many patients with genetically confirmed diagnosis carrying mutations in well-established HSP genes. Our results are in accordance with smaller studies finding prolonged CMCT to legs in the majority of cases and affection of arms in up to 32% [8-10]. The results are consistent with a length-dependent neurodegenerative process.

Conversely, CMCT to legs was normal in about 27% of HSP patients despite unequivocal clinical signs of corticospinal tract affection. This may be explained by a primarily axonal type of damage seen in several subtypes of HSP or alternatively by a selective affection of thinner motoneurons and other motor pathways (i.e. reticulo-spinal or vestibulo-spinal tracts) that spares the fastest conducting thick fibres investigated by transcranial magnetic stimulation.

Additionally, we show that HSP in many cases is not restricted to the motor system but affects the sensory system as well. Abnormal cortical potentials in 38% of tibial nerve SSEPs prove sensory system involvement in a substantial portion of HSP patients. Similarly, sensory NCS were abnormal in about one third of HSP patients. Previous studies included only small numbers of patients and led to variable results [9,12-14].

Here we screened a large HSP cohort that was not selected for clinical signs of peripheral neuropathy and found involvement of peripheral motor nerves in 44% of patients. Tibial nerves were more frequently impaired than ulnar nerves reflecting the more severe affection of longer motor axons to the legs. The affection of upper and lower motoneurons may be of special importance in the differential diagnosis between HSP and amyotrophic lateral sclerosis (ALS). Although we did not perform electromyography (EMG) and cannot differentiate between neuronal and axonal damage, it becomes clear that the combination of upper and lower motor tract affection *per se* is not indicating ALS but is a rather frequent constellation in HSP as well. Recent data suggest a continuum in motoneuron disorders; several genes can underlie different phenotypes, resembling ALS, HSP and HM(SA)N respectively (reviewed in [19]), e.g. *Alsin* (ALS2) [20-22], *Senataxin* (ALS4) [23,24], *NIPA1* (SPG6, NIPA1 repeat expansions associated with ALS) [25,26], *BSCL2* (SPG17, HMSN V) [27-29], *Atlastin-1* (SPG3, HSN-I) [30-32], *KIF1A* (SPG 30, HSAN-II) [33,34], and *REEP1* (SPG31, dHMN-V) [35,36].

Electrophysiologically, there is an interrelation between central and peripheral damage. This could indicate a common length-dependent disease mechanism in several long fibre tracts. Hereby, electrophysiological analyses may provide a window into pathomechanisms that alter conduction parameters in long fibre tracts in a genotype specific manner.

The affection of long fibre tracts is also seen in neuropathological examinations. In pure HSP forms degeneration of the corticospinal tract and the posterior column was compatible with a dying-back axonopathy [37-39]. This result was also confirmed in genetically proven cases of *SPG4*[40,41]. In complicated forms pathology shows more widespread neurodegeneration also affecting the thalamus, brainstem nuclei and the cerebellum [42].

Disturbance of axonal transport as in SPG4 and SPG10 with mutations in Spastin affecting microtubule severing and in KIF5A affecting the motor of anterograde axonal transport may go along with an axonal type of conduction disturbance [41,43,44]. In accordance with this pathophysiological assumption and previous studies [11,15], in our cohort of *SPG4* patients MEPs to the arms were normal. As a rule of thumb, massively elongated CMCT (arms ≥ 15.0 ms, legs ≥ 25.0 ms) argue against a *SPG4* genotype. But even within the group of *SPG4* patients a considerable variability of CMCTs with reduced amplitudes and prolonged latencies were reported [45]. On the basis of the results in two *SPG4* families a role of the mutation type was proposed [16]. In our large cohort of 35 *SPG4* patients MEP latencies in patients with *SPG4* missense mutations were significantly shorter in comparison to *SPG4* splice site mutations, premature stop codons or in-frame exon deletions.

Our data represent a cross-sectional analysis. It will be interesting to learn about the dynamics of electrophysiological abnormalities in HSP. At present, it remains open to speculation whether motor and sensory, central and peripheral systems are affected at the same time and whether there is a linear or a non-linear progression of electrophysiological abnormalities. Longitudinal studies will help to assess electrophysiological parameters as potential biomarkers of the neurodegenerative process. Given the clinical variability of spasticity with temperature and seasons as well as symptomatic treatment, and given the correlation of disease severity and MEP abnormalities demonstrated in this study, electrophysiological parameters may become important progression markers in upcoming interventional trials.

## Conclusions

HSP is considered an upper motoneuron disease primarily affecting the longest fibres to the legs. Our clinical and electrophysiological analysis suggests a more widespread involvement of motor and sensory tracts in the central and peripheral nervous system. Pronounced elongation of MEPs favours against the most common form of HSP, *SPG4*. As electrophysiological measures are independent of symptomatic treatment they might become important progression markers in upcoming HSP trials.

### Availability of supporting data

The data sets supporting the results of this article are included within the article.

## Abbreviations

ADM: Abductor digiti minimi; AH: Abductor hallucis; ALS: Amyotrophic lateral sclerosis; CMAP: Compound motor action potential; CMCT: Central motor conduction time; DML: Distal motor latency; EMG: Electromyography; HSP: Hereditary spastic paraplegia; MEP: Motor evoked potential; ms: Millisecond; NCS: Nerve conduction study; NCV: Nerve conduction velocity; PMCT: Peripheral motor conduction time; SNAP: Sensory nerve action potential; SNCV: Sensory nerve conduction velocity; SPRS: Spastic Paraplegia Rating Scale; SSEP: Somato-sensory evoked potential; TMCT: Total motor conduction time; VLCFA: Very long chain fatty acids.

## Competing interests

LS received research grants of the Deutsche Forschungsgemeinschaft (SCHO754/4-1 and SCHO754/5-1), grants of the German Research Council (BMBF) to Leukonet (01GM0644) and mitoNET (01GM0864), an E-RARE grant to EUROSCAR (01GM1206) and an FP7 grant to NeurOmics (305121). He further received funding from the HSP-Selbsthilfegruppe Deutschland eV.

## Authors’ contributions

RS and LS designed the study. KNK, RS, SK, SO, CF and LS acquired, analysed and interpreted the data. KNK and IL-S performed the statistical analysis. KNK and LS drafted the manuscript. RS, SK, OK, CK an IL-S revised the manuscript critically for important intellectual content. All authors read and approved the final manuscript.
